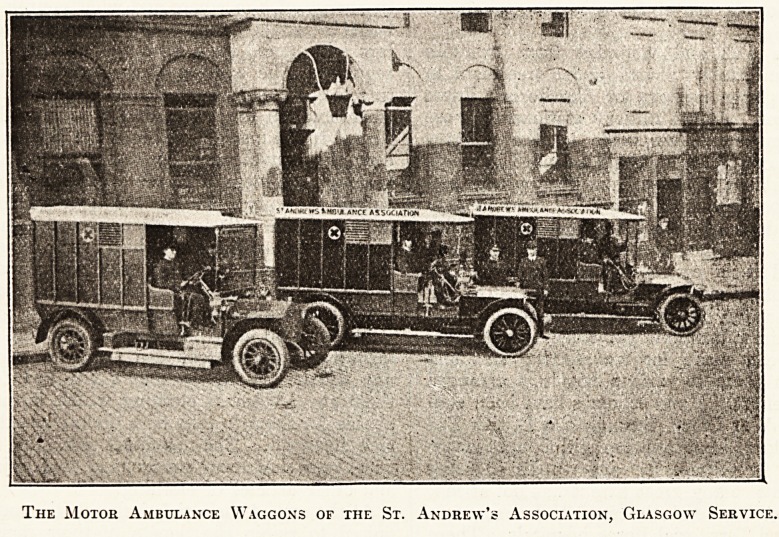# Types of Motor Ambulance Waggons

**Published:** 1914-10-03

**Authors:** 


### TYPES OF MOTOR AMBULANCE WAGGONS.

### The St. Andrew's Association's Models.

In our issue last week, p. 698, we referred to the good ambulance work which is being done in Glasgow by the St. Andrew's Ambulance Association. We now publish illustrations of these waggons, including one of the latest type.

All the Association's motor waggons are fitted with pneumatic tyres, with twin rear detachable rims. The bodies, which have been built specially to the Association's design, are of light construction, the total weight of the waggons, with one exception, being about 33 cwt. The older type is fitted with a spring bed or couch specially made by the Association, resting on small sash rollers. This arrangement enables the couch to be pushed in on the floor of the waggon and a bed-stretcher fixed to the side of the waggon body, which can be suspended by means of straps and hooks from the roof when required. There is also room for two sitting patients, in addition to the ambulance attendant.

### The Equipment and Accommodation.

The new type of waggon, of which two have just been brought into use, is fitted with the spring bed or couch, and with a cushioned seat which can take five sitting-up patients, or can be converted into an additional bed. One of these waggons has no hanging stretcher, but the other, which has a 37.2 Renault chassis, and is of a particularly large and commodious type, is fitted with two hanging stretchers. This waggon, which was given to the Association during the present national emergency, is probably the most perfect type of ambulance waggon in the United Kingdom. It can carry four patients lying down and three sitting up, or two lying down and seven sitting up, in addition to the attendant and driver.

The waggons are all fitted with lockers for the storage of ambulance material and appliances.

### Urgent Need for Motor-Ambulances.

The above facts are of especial interest at the present moment in view of the fact that another aspect of the transport question is vividly brought forward by a correspondent of the *Times*, who urges the imperative need of at least seventy motor-ambulances. Up to September 17, he says, the wounded were brought from the clearing-hospitals on a bed of straw in motor-lorries (he describes the process as an eye-witness in France), sometimes for a distance of twenty miles. "At present," he adds, "about fifty motor-ambulances have arrived in this country, but quite a number of them are needed at each station to carry wounded from the train to the hospital, and less than thirty

St. Andrew's Ambulance Association's Latest Waggon.

The Motor Ambulance Waggons of the St. Andrew's Association, Glasgow Service.

will be able to do duty at the Front. They need there at least 100, and even more, if possible." His appeal to the Red Cross Society will surely not fall on deaf ears, and we hope that the Society will not lose a moment in using some of the money at their disposal for providing at least seventy motor-ambulances which are asked for. The wounded and the doctors at the front are both sadly handicapped by this weak link in the chain of transport, which causes much avoidable suffering, and requires only money and promptitude to be put right.

## Figures and Tables

**Figure f1:**
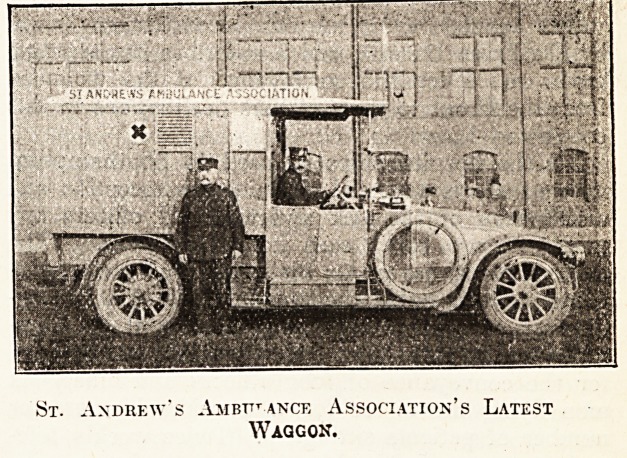


**Figure f2:**